# Genotypic and phenotypic characterization of *Escherichia coli* isolated from mollusks in Brazil and the United States

**DOI:** 10.1002/mbo3.738

**Published:** 2018-10-11

**Authors:** Marília Miotto, Sylvia A. Ossai, Joan E. Meredith, Clarissa Barretta, Airton Kist, Elane S. Prudencio, Cleide R. W. Vieira, Salina Parveen

**Affiliations:** ^1^ Department of Food Science and Technology Federal University of Santa Catarina Florianopolis Santa Catarina Brazil; ^2^ Food Science and Technology Program University of Maryland Eastern Shore Princess Anne Maryland; ^3^ Laboratory of Computational and Applied Statistics, Department of Mathematics and Statistics State University of Ponta Grossa Ponta Grossa Parana Brazil

**Keywords:** *Escherichia coli*, pulsed‐field gel electrophoresis, resistance, serogrouping, shellfish

## Abstract

The aim of this study was to determine the serogroups, antimicrobial resistance and genetic diversity of *Escherichia coli* isolates from samples of bivalve mollusks collected along Santa Catarina coast, Brazil, and from the Chesapeake Bay, Maryland, USA. One hundred forty‐one *E. coli* isolates were characterized for serogroups with 181 specific O antisera and antimicrobial susceptibility using the disk diffusion method. The genetic diversity was assessed using pulsed‐field gel electrophoresis (PFGE). The results showed that among the isolates, 19.9% were classified as multi‐drug resistant (MDR) and resistance was most frequently observed to cephalothin, nitrofurantoin, and ampicillin. The predominant serogroups were O6, O8, and O38. Some serogroups were recognized as pathogenic *E. coli*. PFGE dendrograms indicated extensive genetic diversity among the isolates. Although characteristics of the *E. coli* isolates were highly variable, it is important to note that *E. coli* belonging to pathogenic serogroups and MDR isolates are present in mollusks of both study areas. This is the first report on the phenotypic and genotypic characterization of *E. coli* from mollusks from Santa Catarina and the Chesapeake Bay that should encourage studies focusing on comparison of isolates across countries.

## 1 INTRODUCTION

The microbiological quality of coastal environments is a major issue, especially in shellfish growing areas where water contamination may exist, with sewage discharges, including sewage outfall, combined sewer overflows, and rainwater discharges (Lee, Panicker, & Bej, 2003; Oliveira, Cunha, Castilho, Romalde, & Pereira, 2011). Enteropathogenic strains of *Escherichia coli* are widely distributed in coastal areas and are causative agents of gastroenteritis in humans after consumption of contaminated seafood (Kanayama et al., 2015). As filter‐feeding organisms, bivalves can concentrate contaminants from the surrounding water including micro‐organisms that can cause several infectious diseases in humans (Brands et al., 2005; Ramos et al., 2014). Moreover, as oysters could be consumed raw or lightly cooked, they are potential vectors for pathogenic *E. coli* (Pereira et al., 2017).

*Escherichia coli* is a Gram‐negative, facultative anaerobic bacterium that is primarily present in the gastrointestinal tract of humans and other endothermic organisms. Although most of these commensal *E. coli* strains are harmless, many are pathogenic and can cause diseases in humans. *E. coli* is an indicator of fecal contamination in food, marine and freshwater environments (Noble, Blackwood, Griffith, McGee, & Weisberg, 2010; Zhang, Wu, Zhang, Lai, & Zhu, 2016) and has also been suggested as a possible indicator to assess the antimicrobial resistance status in environmental settings (Berendonk et al., 2015).

The presence of pathogenic strains of *E. coli* in seafood is a public health concern and may lead to serious health risks to consumers (Costa, 2013). Consequently, authorities in various countries, such as Brazil (Brazil, 2012), the United States (FDA, 2015), and the European Union (EU, 2004), have established regulatory limits and monitoring programs using *E. coli* counts, fecal coliform levels of bivalves, or fecal coliform levels of bivalve growing areas. Shiga toxin‐producing *E. coli* (STEC), especially *E. coli* O157:H7, has been widely implicated in outbreaks of foodborne illnesses (CDC, 2018). However, new evidence suggests that non‐O157 isolates belonging to the serogroups O26, O45, O91, O103, O104, O111, O113, O121, O128, O145, and O146 also cause significant human illnesses (Frank et al., 2011; Mellmann et al., 2009; Shao, Li, Jia, Lu, & Wang, 2003; Stritt et al., 2013; USDA, 2012).

*Escherichia coli* has been detected in bivalves from different parts of the world, and some pathogenic serotypes have been isolated (Balière, Rincé, Thevenot, & Gourmelon, 2015; Bennani et al., 2011; Gourmelon et al., 2006; Guyon et al., 2000) which enforces the importance of the study of *E. coli* isolates from mollusks. *E. coli* has been isolated from bivalve mollusks in different studies in Santa Catarina coast in Brazil (Pereira, Nunes, Nuernberg, Schulz, & Vieira Batista, 2006; Ramos et al., 2012) and in other states in Brazil (Forcelini, Kolm, & Absher, 2013; Ribeiro et al., 2016). In the United States, Montazeri et al. (2015) reported *E. coli* isolated from oysters. However, no study was conducted to characterize *E. coli* recovered from mollusks by genotypic and phenotypic methods in the United States and in Brazil.

Serotyping is an important tool for the differentiation of *E. coli* strains, but it does not comprehensively characterize a strain. In recent years, various genotyping methods such as pulsed‐field gel electrophoresis (PFGE) have been used to differentiate *E. coli* and determine the genetic relationships of strain. PFGE is considered as the gold standard method because of its high level of discrimination and it has also been reported that this technique could be the most discriminatory genotypic method to provide a reproducible DNA fingerprinting (Zhang et al., 2016).

Antimicrobial resistance (AMR) is another tool for characterization of bacterial isolates. With the wide use of antimicrobials in humans and in the environment, AMR *E. coli* have been reported from different sources and countries (Dou et al., 2016; Kao et al., 2016; Rabbia et al., 2016; Zhang et al., 2016). The presence of resistant organisms in the environment is an emerging concern around the world (Watkinson, Micalizzi, Graham, Bates, & Costanzo, 2007), and since AMR bacteria can accumulate in bivalve mollusks (Barkovskii, Green, & Hurley, 2010), it has been suggested that bivalves may be useful in assessing environmental contamination by AMR bacteria (Berendonk et al., 2015; Rees et al., 2015).

Until now, little information is available on the genotypic and phenotypic characteristics of *E. coli* isolated from samples of mollusks in Santa Catarina, Brazil, and the Chesapeake Bay, Maryland, USA, and no data have been published regarding the genetic relatedness between *E. coli* isolates from Brazil and the United States. Considering that both Santa Catarina and the Chesapeake Bay are known for mollusk production (NOAA, 2012; Santos, Marchiori, & Della Giustina, 2017), research is important to improve the knowledge about the *E. coli* strains in samples from those harvesting areas. Since bivalves can accumulate micro‐organisms, including *E. coli*, present in surrounding waters by their filter‐feeding activities and may present a risk to public health, studies on the characterization of those strains should be addressed. The aim of this study was to determine the serogroups, antimicrobial resistance and genetic diversity of *E. coli* isolates recovered from mussels and oysters collected in two distant global regions.

## 2 MATERIAL AND METHODS

### 2.1 Collection of samples (mussels and oysters)

In Santa Catarina, South Brazil coast, a total of 100 samples were collected from 10 different localities (São Francisco do Sul, Balneário Barra do Sul, Penha, Balneário Camboriu, Bombinhas; Porto Belo, Gov. Celso Ramos, Florianópolis, São José and Palhoça) from January to July of 2015 in an interval of three weeks. They were comprised of 40 samples of oysters (*Crassostrea gigas* and *Crassostrea rhizophorae*) and 60 samples of mussels (*Perna perna*). Each sample was comprised of 12 mollusks. In the Chesapeake Bay, Maryland, USA, a total of 18 samples of oysters (*Crassostrea virginica*) were taken from five localities (Oxford, Manokin, Chester, Broad Creek, and Tangier Sound) during October and November of 2015. Sampling frequency was every 3 weeks, and each sample was comprised of 12 mollusks. Mussel samples were collected only from Santa Catarina coast because mussels represent 82% of total bivalve mollusk production while oysters represent 15% (Santos et al., 2017).

The samples in Brazil were collected by hand, and in the United States, an oyster dredge was used. Immediately after harvesting, oyster samples were bagged and placed in insulated chests. Bubble wraps were placed between the oyster bags and ice bags to prevent direct contact with ice and water. The shipping temperature was monitored by data loggers (ACR Systems, Inc., Data Logger Store, Contoocook, NH, USA) to ensure that it was maintained between 2 and 10°C. All microbiological analyses were initiated within 4 hr of sample collection.

### 2.2 Microbiological analysis

All 118 samples were examined quantitatively for *E. coli* by a five tube most probable number (MPN) method using minerals modified glutamate broth (MMGB) (Oxoid Ltd, Basingstoke, Hampshire, UK) and the chromogenic medium Tryptone Bile 5‐bromo‐4‐chloro‐3‐indolyl‐β‐d‐glucuronide agar (TBX) (Oxoid), in accordance with ISO 16649‐3 method (ISO, 2015). The results were given as the number of *E. coli* in MPN/100 g. The lowest detectable concentration of *E. coli* when applying this method was 20 MPN/100 g.

### 2.3 Antimicrobial susceptibility testing

The susceptibility to antimicrobials was tested by the disk diffusion method according to the guidelines published by the Clinical and Laboratory Standards Institute (CLSI, 2012) on Mueller‐Hinton agar (Oxoid). Bacterial suspension (pure culture) was adjusted to a 0.5 McFarland standard and tested against the following antimicrobials: amikacin (AN, 30 μg), amoxicillin/clavulanic acid (AMC, 20/10 μg), ampicillin (AM, 10 μg), cefepime (FEP, 30 μg), cefoxitin (FOX, 30 μg), ceftriaxone (CRO, 30 μg), cephalothin (KF, 30 μg), chloramphenicol (C, 30 μg), ciprofloxacin (CIP, 5 μg), gentamicin (GM, 10 μg), nalidixic acid (NA, 30 μg), nitrofurantoin (FM, 300 μg), norfloxacin (NOR, 10 μg), tetracycline (TE, 30 μg), and trimethoprim/sulfamethoxazole (SXT, 1.25/23.75 μg). Antimicrobial disks were obtained from Becton, Dickinson and Company (Sparks, MD, USA) and Oxoid. *E. coli* ATCC 25922 and *Pseudomonas aeruginosa* ATCC 27853 (American Type Culture Collection, Manassas, VA, USA), strains were used as controls in each assay (Pagadala et al., 2012).

*Escherichia coli* isolates were classified as susceptible, intermediate resistant, or resistant according to the CLSI criteria for Enterobacteriaceae (CLSI, 2014). A strain was considered multi‐drug resistant (MDR) when demonstrating resistance to three or more antimicrobial classes (Schwarz et al., 2010).

*Escherichia coli* isolates were tested for AMR index with the intent to find a correlation with pollution sources since this method was shown to be useful in differentiating human from nonhuman pollution sources in previous studies (Watkinson et al., 2007; Webster et al., 2004). The AMR indices were calculated as follows: isolate AMR index = no. of antimicrobials to which the isolate was resistant/total no. of antimicrobials tested (Parveen et al., 1997).

### 2.4 Serogrouping

All *E. coli* isolates were referred to *E. coli* Reference Center located at Pennsylvania State University, Pennsylvania, USA, for serogrouping. The isolates were serogrouped for “O” antigen according to the methodology described by Orskov, Orskov, Jann, and Jann (1977). The method used slide agglutination with O antigen‐specific antisera, employing O1‐O187 antisera (with the exceptions of O31, O47, O67, O72, O94, O122) with a total of 181 antisera. Serogroups associated with the five categories of gastrointestinal pathogenic *E. coli*, which include enteropathogenic *E. coli* (EPEC), enterotoxigenic *E*. *coli* (ETEC), enteroinvasive *E*. *coli* (EIEC), enteroaggregative *E*. *coli* (EAEC), and enterohemorrhagic *E. coli* (EHEC) were considered as pathogenic serogroups.

### 2.5 Molecular typing

Genetic diversity of the isolates was assessed using PFGE according to a standard protocol developed by the United States Centers for Disease Control and Prevention (CDC) for *E. coli*. Briefly, agarose plugs were prepared with *E. coli* cell suspension, lysed with proteinase K and digested using *Xba*I enzyme. DNA fragments were separated on 1.5% agarose gel by electrophoresis on a CHEF DR‐III system (Bio‐Rad Laboratories, USA). The gel was stained with ethidium bromide (40 mg/ml) and then de‐stained with deionized water and visualized with ultraviolet light. A molecular size standard (*Salmonella* enterica serotype Braenderup H9812) was used.

The results were evaluated using Bionumerics software (AppliedMaths, Austin, TX, USA). PFGE patterns were established based on the number and arrangement of fragments and computationally based on the levels of relatedness using the Dice similarity coefficient and unweighted pair group method using arithmetic averages (UPGMA) with 1.5% optimization and 1.5% tolerance and Pearson correlation coefficient (0.5%). A phylogenetic dendrogram was constructed based on PFGE fingerprint profiles. Isolates sharing at least 80% similarity were considered genetically related, and those sharing 100% similarity were classified as clones (Balière, Rincé, Blanco, et al., 2015; Kao et al., 2016; Rabbia et al., 2016).

### 2.6 Statistical analysis

The distribution of pathogenic serogroups between the sample sites was compared using the chi‐square test. A two proportion test was conducted to evaluate the incidence of MDR isolates and pathogenic serogroups between isolates from Brazil and the United States. The *E. coli* counts and the incidence of pathogenic serogroups were compared with the Mann–Whitney *U* test and logistic regression. Logistic regression analysis was also applied to evaluate relationships between pathogenic serogroups and MDR profiles; pathogenic serogroups and sites; pathogenic serogroups and *E. coli* counts and MDR profiles. These analyses were applied separately to Brazil and US isolates. A *p* value <0.05 was considered statistically significant. The statistical analysis was performed using Minitab Software, version 16.2.4.0 (Minitab Inc., State College, PA, USA).

## 3 RESULTS

### 3.1 Prevalence and concentration of *E. coli*

All samples (100%) collected from Brazil (BR) and eleven (61%) of US samples were positive for *E. coli*, the counts varied widely, with concentrations ranging from 20 to 18,000 MPN/100 g among samples from Brazil and among samples from the United States, the concentrations ranged from <20 to 130 MPN/100 g. Forty‐nine percent of the samples from Brazil presented a concentration less than 230 MPN/100 g, 43% contained between 230 and 4,600 *E. coli* MPN/100 g and eight percent presented concentration higher than 4,600 MPN/100 g. In 54% of the oyster samples from the United States that were positive for *E. coli,* the bacterial counts were 20 MPN/100 g. No *E. coli* was found in samples from Manokin site.

A total of 141 *E. coli* isolates were recovered from samples collected in Brazil (*n* = 100) and in the United States (*n* = 41) for genotypic and phenotypic characterization. One typical blue colony of β‐glucuronidase‐positive *E. coli* was selected from each positive sample in Brazil (60 isolates from mussels and 40 isolates from oysters). Three to five colonies were selected from each positive sample in the United States due to small sample size.

### 3.2 Antimicrobial susceptibility testing

*Escherichia coli* isolates were tested for susceptibility to 15 antimicrobial agents of veterinary and human health significance. Of the total number of isolates, 83% (*n* = 117) were resistant to at least one antimicrobial agent and 19.9% (*n* = 28) were classified as MDR. Most of the *E. coli* isolates, from the United States and Brazil, were susceptible to cefepime (99.3%), norfloxacin (97.2%), chloramphenicol (95.7%) and trimethoprim/sulfamethoxazole (91.5%). Resistance was most frequently observed in cephalothin (78.0%), nitrofurantoin (21.3%) and ampicillin (19.9%) and intermediate resistance in ampicillin (50.4%), nitrofurantoin (46.8%), and amoxicillin/clavulanic acid (44.0%) (Table 1).

**Table 1:** Percentages of antimicrobial resistance and intermediate resistance determined by the disk diffusion method in *Escherichia coli* isolates from mollusks samples from Brazil and the United States

Antimicrobial	Brazil (*n* = 100)		United States (*n* = 41)		Total (*n* = 141)	
	Resistant	Intermediate	Resistant	Intermediate	Resistant	Intermediate
KF	73.0	26.0	90.2	9.8	78.0	21.3
FM	12.0	46.0	43.9	48.8	21.3	46.8
AM	19.0	49.0	22.0	53.7	19.9	50.4
TE	16.0	2.0	0	0	11.3	1.4
FOX	0	4.0	31.7	12.2	9.2	6.4
SXT	9.0	2.0	0	2.4	6.4	2.1
NA	6.0	24.0	4.9	22.0	5.7	23.4
AMC	0	40.0	19.5	53.7	5.7	44.0
CRO	5.0	25.0	2.4	17.1	4.3	22.7
AN	2.0	17.0	0	43.9	1.4	24.8
GM	1.0	6.0	2.4	24.4	1.4	11.3
CIP	1.0	11.0	0	24.4	0.7	14.9
C	1.0	5.0	0	0	0.7	3.5
FEP	1.0	0	0	0	0.7	0
NOR	1.0	3.0	0	0	0.7	2.1

Note

AM: ampicillin; AMC: amoxicillin/clavulanic acid; AN: amikacin; C: chloramphenicol; CIP: ciprofloxacin; CRO: ceftriaxone; FEP: cefepime; FM: nitrofurantoin; FOX: cefoxitin; GM: gentamicin; KF: cephalothin; NA: nalidixic acid; NOR: norfloxacin; SXT: trimethoprim/sulfamethoxazole; TE: tetracycline.

Thirty‐three antimicrobial resistance profiles were observed among *E. coli* isolates recovered from mollusks from Brazil and the United States. The most predominant resistance profiles were KF; KF‐FM; KF‐AM, with 53 (37.6%), 12 (8.5%), and 7 (5.0%) isolates in each, respectively, and five antimicrobial profiles were shared among *E. coli* isolates from Brazil and the United States: KF‐FM‐NA; KF‐GM‐FM; KF‐AM; KF‐FM; KF (Table 2).

**Table 2:** Antimicrobial resistance profiles determined by the disk diffusion method of *Escherichia coli* isolates from mollusks samples from Brazil and US samples

Resistance profile	Number (%) of isolates in samples from:		
	Brazil (*n* = 100)	United States (*n* = 41)	Total (*n* = 141)
KF‐AM‐SXT‐TE‐NOR‐NA‐C^a^	1 (1.0)	0	1 (0.7)
KF‐AM‐SXT‐TE‐CRO‐FM^a^	1 (1.0)	0	1 (0.7)
KF‐AM‐SXT‐TE‐CRO^a^	1 (1.0)	0	1 (0.7)
KF‐AM‐SXT‐TE‐FM^a^	1 (1.0)	0	1 (0.7)
KF‐AM‐FM‐FOX‐AMC^a^	0	2 (4.9)	2 (1.4)
KF‐AM‐FM‐TE‐NA^a^	1 (1.0)	0	1 (0.7)
KF‐AM‐FM‐TE^a^	2 (2.0)	0	2 (1.4)
KF‐AM‐FOX‐AMC^a^	0	4 (9.8)	4 (2.8)
KF‐AM‐FOX‐CRO	0	1 (2.4)	1 (0.7)
KF‐SXT‐AM‐TE^a^	1 (1.0)	0	1 (0.7)
KF‐SXT‐TE‐CRO^a^	1 (1.0)	0	1 (0.7)
KF‐FM‐FOX‐AMC^a^	0	2 (4.9)	2 (1.4)
KF‐FM‐NA^a^	1 (1.0)	2 (4.9)	3 (2.1)
KF‐AM‐TE^a^	2 (2.0)	0	2 (1.4)
KF‐AM‐FOX	0	1 (2.4)	1 (0.7)
KF‐AM‐AN^a^	1 (1.0)	0	1 (0.7)
KF‐GM‐FM^a^	1 (1.0)	1 (2.4)	2 (1.4)
KF‐CRO‐AM	1 (1.0)	0	1 (0.7)
CRO‐TE‐NA^a^	1 (1.0)	0	1 (0.7)
SXT‐TE‐AM^a^	1 (1.0)	0	1 (0.7)
SXT‐TE‐KF^a^	1 (1.0)	0	1 (0.7)
KF‐AM	6 (6.0)	1 (2.4)	7 (5.0)
KF‐FM	2 (2.0)	10 (24.4)	12 (8.5)
KF‐CIP	1 (1.0)	0	1 (0.7)
KF‐AN	1 (1.0)	0	1 (0.7)
KF‐FOX	0	2 (4.9)	2 (1.4)
KF‐NA	2 (2.0)	0	2 (1.4)
KF‐TE	2 (2.0)	0	2 (1.4)
KF‐SXT	1 (1.0)	0	1 (0.7)
FM‐FOX	0	1 (2.4)	1 (0.7)
FM‐FEP	1 (1.0)	0	1 (0.7)
FM	2 (2.0)	0	2 (1.4)
KF	42 (42.0)	11 (26.8)	53 (37.6)

Note

AM: ampicillin; AMC: amoxicillin/clavulanic acid; AN: amikacin; C: chloramphenicol; CIP: ciprofloxacin; CRO: ceftriaxone; FEP: cefepime; FM: nitrofurantoin; FOX: cefoxitin; GM: gentamicin; KF: cephalothin; NA: nalidixic acid; NOR: norfloxacin; SXT: trimethoprim/sulfamethoxazole; TE: tetracycline.

a MDR profiles.

The lowest AMR index observed was 0, and the highest was 0.47. The most frequent AMR index was 0.07 (44%) and 0.13 (17%) among Brazil isolates and 0.13 (34.2%) and 0.07 (26.8%) among US isolates.

Among isolates from Brazil, most of them were susceptible to cefepime (99%), norfloxacin (96%), cefoxitin (96%), and chloramphenicol (94%); on the other hand, cephalothin (73%), ampicillin (19%), tetracycline (16%), and nitrofurantoin (12%) were the antimicrobials with the higher percentages of resistant strains. Seventy‐eight percent of the isolates were resistant to at least one antimicrobial agent, and 17% exhibited MDR. In total, there were 26 antimicrobial resistance profiles observed among Brazilian isolates.

Regarding the isolates harvested in the United States, all of them were susceptible to chloramphenicol, cefepime, norfloxacin, and tetracycline and 90.2% were resistant to cephalothin, 43.9% were resistant to nitrofurantoin, 31.7% to cefoxitin, and 22% were resistant to ampicillin. Ninety‐five percent of the isolates were resistant to at least one antimicrobial agent, and 26.8% were classified as MDR (Table 1). There were a total of 12 antimicrobial resistance profiles observed among US isolates (Table 2).

### 3.3 Serogrouping

A total of 141 *E. coli* isolates were serogrouped, and 81% were typeable. The typed isolates displayed 49 different serogroups that are shown in Table 3. Forty‐four and 11 different serogroups were found between *E. coli* isolates from Brazil and the United States, respectively. Six serogroups (O6, O9, O25, O54, O88, and O113) were shared by isolates from Brazil and the United States. Some *E. coli* isolates possess O‐serogroups (O5, O6, O7, O8, O15, O25, O45, O86, O88, O91, O112, O113, O126, O128, O146, O159) that have been recognized as pathogenic *E. coli*.

**Table 3:** O‐serogroups of *Escherichia coli* isolates from mollusks samples from Brazil and US samples

O‐Serogroups	Number (%) of *E. coli* isolates	
	Brazil (*n* = 100)	USA (*n* = 41)
6	7 (7)	2 (4.9)
9	2 (2)	4 (9.8)
25	1 (1)	2 (4.9)
54	1 (1)	2 (4.9)
88	2 (2)	1 (2.4)
113	1 (1)	4 (9.8)
8	17 (17)	0
20	0	3 (7.3)
21	4 (4)	0
38	0	11 (26.8)
37	2 (2)	0
45	2 (2)	0
85	2 (2)	0
86	2 (2)	0
91	2 (2)	0
93	0	2 (4.9)
126	2 (2)	0
139	2 (2)	0
159	2 (2)	0
175	3 (3)	0
180	2 (2)	0
(107, 177)^a^	1 (1)	0
(17, 73, 77, 106)^a^	4 (4)	0
(5, 11, 12, 15, 23, 33, 40, 59, 75, 82, 96, 105, 112, 128, 146, 148, 150, 163, 166, 176)^b^	20 (20)	0
(7, 64)^c^	0	2 (4.9)
M	7 (7)	3 (7.3)
N	12 (12)	5 (12.2)

Note

M: reacted with antisera of several serogroups; N: did not react with any known O groups.

a Serogroups that were identified in the same isolate.

b Serogroups that contained only one isolate (Brazil).

c Serogroups that contained only one isolate (USA).

The most common serotype among US isolates was O38 (26.8%), followed by O9 (9.8%), O113 (9.8%), and O20 (7.3%). Other serotypes included O6, O25, O54, O93 with 4.9% of frequency and O7, O64, and O88 with 2.4% of the isolates (Table 3). Among *E. coli* isolates from Brazil, the predominant serogroups were O8 (17%), O6 (7%), O17, O21, O73, O77, and O106 (4%) (Table 3).

Ten isolates (seven from Brazil and three from the United States) were classified as M (reacted with antisera of several serogroups and could not be determined). In addition, 17 isolates (12 from Brazil and five from the United States), did not react with any known O groups, being classified as N.

### 3.4 Molecular typing

**Figure 1:**
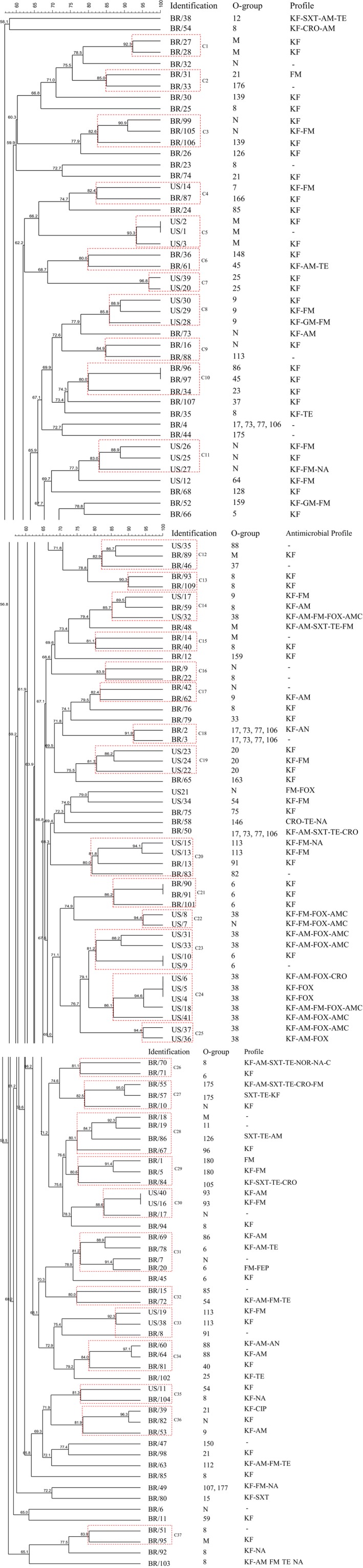
Dendrogram of PFGE patterns of *Escherichia coli* isolated from mollusks harvested in Brazil and the United States. AM: ampicillin; AMC: amoxicillin/clavulanic acid; AN: amikacin; C: chloramphenicol; CIP: ciprofloxacin; CRO: ceftriaxone; FEP: cefepime; FM: nitrofurantoin; FOX: cefoxitin; GM: gentamicin; KF: cephalotin; NA: nalidixic acid; NOR: norfloxacin; SXT: trimethoprim/sulfamethoxazole; TE: tetracycline; (−): not resistant; M: reacted with antisera of several serogroups; N: did not react with any known O groups; C1 to C37: identification of the clusters considering 80% similarity

Three dendrograms were constructed and evaluated: one containing the isolates from Brazil and the United States together (Figure 1); one dendrogram with the isolates from Brazil (not shown); and a third dendrogram including the isolates from the United States (not shown). A total of 134 PFGE banding patterns were generated from 141 *E. coli* recovered from oysters and mussels, indicating extensive genetic diversity among the isolates from Brazil and the United States. However, at an 80% similarity level, 37 clusters were identified and, in six clusters Brazil and US isolates grouped together with between two and four isolates in each. With 100% similarity**,** six clusters were identified and the US and Brazil isolates are clearly separated (Figure 1).

Among isolates from Brazil, a total of 98 PFGE banding patterns were generated from 100 *E. coli* isolates, indicating extensive genetic diversity among these isolates too. Considering 80% similarity, 65 subgroups were identified with 24 clusters with between one and four isolates in each. Nevertheless, at a 100% similarity level, two clusters were identified with two isolates considered clones in each.

Regarding the dendrogram constructed with *E. coli* isolates from the United States, a total of 36 patterns were generated from 41 *E. coli* isolates. With 80% similarity, PFGE patterns grouped into 14 clusters, and at a 100% similarity, four clusters were clearly differentiated with between two and three isolates in each. These clusters showed concordance with the serogroups, isolation site, and antimicrobial profile, with few exceptions.

## 4 DISCUSSION

Since *E. coli* is a well‐known indicator of recent fecal contamination, and it was demonstrated that oysters can bioaccumulate fecal coliforms to a concentration four times greater than surrounding water (Burkhardt & Calci, 2000), isolation and characterization of *E. coli* from bivalve mollusks are essential. Moreover, some serogroups of *E. coli* can be highly pathogenic to humans (Ramos et al., 2012) and the development of antibacterial resistance is an important public health issue (Aitken, Dilworth, Heil, & Nailor, 2016). The search for antimicrobial‐resistant bacteria, their phylogenetic lineages and serogrouping is relevant and necessary. This is the first reported study on the compared characterization of *E. coli* isolates from mollusks from Brazil and the United States. Samples were positive for *E. coli* in both locations, but only 8% of samples in Brazil exceeded the legislation limit of 4,600 MPN/100 g (Brazil, 2012). The *E. coli* level in 33% of samples in the United States was 20 MPN/100 g. In the United States, the fecal coliform counts in the harvesting area’s water shall not exceed 40 MPN/100 ml. Since it has been reported that the level of fecal coliforms/*E. coli* is higher in oysters than surrounding water samples (Parveen et al., 1997), we may suggest that the counts in water samples were in accordance with the legislation limit.

Of the 15 tested antimicrobials, *E. coli* were most resistant to cephalothin, nitrofurantoin, and ampicillin (Table 1). These results are consistent with previous findings (Dou et al., 2016; Parveen et al., 1997; Rees et al., 2015; Ryu, Lee, et al., 2012). Authors have reported that *E. coli* isolates are generally resistant to antimicrobials which have been in use the longest time in human and veterinary medicine, like nitrofurantoin, ampicillin, and tetracycline (Dou et al., 2016). Our study revealed that a high percentage (31.7%) of *E. coli* isolates from the United States were resistant to cefoxitin while none of the Brazilian isolates were resistant. On the other hand, 16% of isolates from Brazil were resistant to tetracycline while no US isolate presented resistance to that antimicrobial (Table 1). This might be due to geographical variation and different practices of antimicrobial use in the two countries.

Based on the AMR indices in the United States, more antimicrobial resistance was observed in samples from Oxford site (mean AMR = 0.18), followed by Tangier Sound (mean AMR index = 0.15), Chester (mean AMR index = 0.15), and Broad Creek (mean AMR index = 0.12). The AMR indices showed that there was more antimicrobial resistance in the samples collected in November, autumn in the United States (mean AMR index = 0.17), and in February, summer in Brazil (mean AMR index = 0.13). The sites with the more resistant bacteria could be influenced by urban sewage discharges in Brazil and by runoff from the agricultural lands in the United States. The different seasons in the two countries that cause influence on the water temperatures (Parveen et al., 2017; Ramos et al., 2012; Raszl, Froelich, Vieira, Blackwood, & Noble, 2016) and also the difference between the sample sizes may explain that observation. Differentiation between human and agricultural sources of *E. coli* strains has been limited; however, the use of AMR index has demonstrated potential for differentiating *E. coli* sources (Parveen et al., 1997; Watkinson et al., 2007; Webster et al., 2004).

Of the total number of isolates, 19.9% (*n* = 28) were MDR strains which is lower than the results observed by Van, Chin, Chapman, Tran, and Coloe (2008) in Vietnam, where 35% of the *E. coli* isolates from shellfish were MDR. Different handling of antimicrobials in this region could explain the different resistance levels. We found that the percentage of MDR isolates among *E. coli* recovered from samples in the United States (26.8%; *n* = 11) was higher than isolates from Brazil (17%; *n* = 17). Nevertheless, no statistical significance was found about the incidence of MDR isolates in Brazil and US samples. Vignaroli et al. (2012) suggested that coastal marine sediment may be a suitable environment for the survival of pathogenic and MDR *E. coli* strains which could explain the incidence of MDR strains in both study areas. Sample size unevenness, considering that up to five isolates from the same sample were studied in US samples, seasonal and geographical variation may have affected the prevalence of MDR strains. The observations from this study do not explain the link between antimicrobial usage and an increase in antimicrobial resistance among *E. coli* isolates, but studies suggested a relationship between the usage of specific antimicrobials and increases in antimicrobial‐resistant bacteria (Ryu, Park, et al., 2012). A study conducted by Rhodes et al., (2000) provided direct evidence that related tetracycline resistance‐encoding plasmids have disseminated between different *Aeromonas* species and *E. coli* and between human and aquaculture environments in distinct locations in England. Results obtained by Furushita et al. (2003) suggest that resistance genes from fish farm bacteria have the same origins as those from clinical strains in a study conducted in Japan.

Is important to note that carbapenem‐resistant and extended‐spectrum β‐lactamase (ESBL)‐producing *E. coli* has become widespread in different reservoirs and represent an emerging public health threat (Nirupamaa et al., 2018; Pulss, Semmler, Prenger‐Berninghoff, Bauerfeind, & Ewers, 2017; Randall et al., 2017). Some studies have also indicated that the transfer of ESBL‐producing bacteria and/or ESBL‐encoding genes to humans could happen *via* the food chain (Ewers, Bethe, Semmler, Guenther, & Wieler, 2012). Studies on carbapenem‐resistant and ESBL in *E. coli* isolates from mollusks and other food matrices should be addressed since they were not included in this work but they play an important role in the studies with AMR.

Diverse serogroups were found in the *E. coli* isolates in this study. Most of the isolates belonged to common serogroups O6, O8, O9, and O38 (Table 3), however, some *E. coli* isolates possess O‐serogroups (O5, O6, O7, O8, O15, O25, O45, O86, O88, O91, O112, O113, O126, O128, O146, O159) that have been recognized as pathogenic and some serogroups have been reported in previous studies. The serotypes O6, O7, and O8 were detected in the present study and were the most frequent serogroups found in a study conducted by Zhang et al. (2016) with ETEC isolates in retail ready‐to‐eat foods in China. These serogroups have been detected in clinical ETEC strains from different countries and were reported to be associated with human ETEC infections (Ansaruzzaman et al., 2007; Konishi et al., 2011; Rodas et al., 2011).

The serogroup O15 that was identified in one *E. coli* isolate recovered from an oyster sample in Brazil was found by Balière, Rincé, Thevenot, et al. (2015) in EPEC *E. coli* isolate from a mussel sample collected from a shellfish‐harvesting site in France and by Regua‐Mangia, Gomes, Vieira, Irino, and Teixeira (2009) in Brazil. The serogroups O5 and O146 recovered from mussel samples in Brazil and the serogroups O113 and O88, found among the *E. coli* isolates from both Brazil and the United States (Table 3), were identified among EPEC isolates recovered from shellfish samples collected in shellfish‐harvesting sites in France (Balière, Rincé, Blanco, et al., 2015). The serogroups O86 (*n* = 2); O126 (*n* = 2) and O128 (*n* = 1) were detected among *E. coli* isolates from Brazil (Table 3). Those serogroups are among the 12 O‐serogroups that have been recognized as EPEC (Meng, LeJeune, Zhao, & Doyle, 2013). The serogroup O86 was found among EAEC strains isolated from children in Rio de Janeiro, Brazil (Regua‐Mangia et al., 2009).

It has been reported that contamination of the environment with *E. coli* strains, such as STEC and EPEC, may occur through the spreading of livestock manure, animal waste on pastures, via wastewaters from slaughterhouses or from treatment plant effluents and by wildlife (Muniesa, Jofre, García‐Aljaro, & Blanch, 2006; Singh et al., 2015). Some serogroups that can cause significant illness (Mellmann et al., 2009; USDA, 2012), the O45 (*n* = 2), O91 (*n* = 2), and O113 (*n* = 1) were detected in *E. coli* isolates recovered from samples from Brazil; also, the serogroup O113 (*n* = 4) was identified in *E. coli* isolates from the United States.

Based on statistical analysis, the presence of pathogenic serogroups was not associated with a specific site of collection, country of study, or incidence of MDR strains. However, in Brazil, the pathogenic serogroups were most frequently observed in mussel samples rather than in oysters (*p* < 0.05). In the United States, at a significance level of 5%, *E. coli* counts were higher in oyster samples where pathogenic serogroups were identified. It cannot be confirmed statistically in samples from Brazil.

The genetic relatedness of the 141 *E. coli* isolates was analyzed by PFGE, and the dendrograms revealed a high degree of genetic diversity (Figure 1). Regarding the six clusters with isolates from Brazil and the United States considered genetically related with 80% similarity (Figure 1), the isolates belonged to different serogroups and no similarity in antimicrobial resistance profile was found, except in isolates that were resistant to cephalothin. Even with only six clusters, a genetic relatedness among Brazil and US isolates was noticed by PFGE. Some previous studies have shown that the global growth of economic activity, tourism, and human migration is leading to more cases of the movement of both diseases and its vectors. Additionally, with the massive increase of world travel, especially by air, opportunities for the spread of pathogenic bacteria, including *Salmonella*,* Campylobacter*,* Shigella*,* Vibrio cholerae,* and *E. coli* have been greatly facilitated (Koornhof, Keddy, & McGee, 2001; Tatem, Rogers, & Hay, 2006). The spread of micro‐organisms across countries was also documented too (Ruiz et al.., 2000). Those observations, and also the food trade between Brazil and the United States (Azevedo, Chaddad, & Farina, 2004), may be considered hypotheses for the presence of genetically related *E. coli* strains in Brazil and the United States as it has been previously demonstrated for *V. cholerae*,* Shigella* species, and enterohemorrhagic *E. coli* using PFGE (Koornhof et al., 2001).

In this study, many tested isolates showed a tendency to cluster based on their date of collection, serogroup, and antimicrobial resistance profile but collected from distant sites which could be observed in six different clusters (data not shown). For US isolates, four clusters were identified at 100% similarity index, we observed that in each cluster the *E. coli* isolates were recovered from the same sample and belonged to the same serogroup with the same antimicrobial susceptibility profile (data not shown).

The genetic diversity was expected in Santa Catarina where mollusks were collected from 10 different sites within 7 months. In the United States, genetic variability was clearly observed too, even though the collection of samples was over a shorter period of time than Brazil. This was also observed in a previous study (Balière, Rincé, Blanco, et al., 2015) with mollusks with fewer collection sites than the present study and also with *E. coli* isolates from other food samples (Zhang et al., 2016). It has been observed that the genetic variability among *E. coli* strains may be due to adaptive mutations that occur, for example, under stress conditions and that leads these strains to selective advantage (Foster, 2005). Furthermore, insertions and deletions in specific regions of the genome enhance the genetic variability and it could be reflected in the PFGE dendrogram (Kaas, Friis, Ussery, & Aarestrup, 2012). These findings could substantiate the genetic variability found in the present study and also the diversity of antimicrobial patterns and serogroups found.

This study contributed to the knowledge of genotypic and phenotypic characteristics of *E. coli* from mollusks samples. Some serogroups found in the present study are among the serogroups that could cause significant human illnesses; however, even the presence of non‐pathogenic *E. coli* in mollusks should alert the public health since this bacterium is recognized as an indicator of fecal contamination. The antimicrobial resistance profiles of *E. coli* isolates from mollusks are a cause of concern, especially considering the MDR strains. Surveillance of environmental samples must be encouraged to comprehensively assess antimicrobial resistance in environmental bacteria. An extensive genetic diversity among the isolates from Brazil and the United States was observed; however, on the other hand, some isolates related were found. Those results encourage surveillance of environmental samples from different countries and perspective studies of genetic relatedness of the isolates.

## CONFLICT OF INTEREST

No conflict of interest declared.

## AUTHORS CONTRIBUTION

MM and SP contributed to the conception and design of the study; CRWV contributed to the conception of the study; MM performed the experiments; JEM, SAO, and CB contributed to the experiments; MM wrote and organized the manuscript; AK conducted the statistical analyses; JEM contributed significantly to the revision of the manuscript; SP contributed significantly to the revision of the manuscript and was responsible for the integrity of the work; SP, ESP, and MM approved the final version of the paper.

## ETHICS STATEMENT

Not required.

## Data Availability

The authors declare that all data are included in the main manuscript.
